# Empathy in primary progressive aphasia: Neural signatures and longitudinal trajectories

**DOI:** 10.1002/alz.71519

**Published:** 2026-05-25

**Authors:** Natacha Cordonier, Sophie Matis, Her Teng, James Carrick, Yun Hwang, Rebekah M. Ahmed, Ramón Landin‐Romero, Olivier Piguet

**Affiliations:** ^1^ Brain and Mind Centre The University of Sydney Camperdown NSW Australia; ^2^ School of Psychology Faculty of Science The University of Sydney Camperdown NSW Australia; ^3^ School of Health Sciences Faculty of Medicine and Health The University of Sydney Camperdown NSW Australia; ^4^ Gosford Hospital Gosford NSW Australia; ^5^ School of Medical Sciences The University of Sydney Camperdown NSW Australia; ^6^ Memory and Cognition Clinic Department of Clinical Neurosciences Royal Prince Alfred Hospital Camperdown NSW Australia

**Keywords:** cortical thickness, empathy, logopenic primary progressive aphasia, longitudinal assessment, nonfluent primary progressive aphasia

## Abstract

**INTRODUCTION:**

Empathy is increasingly recognised as compromised in neurodegenerative disorders. However, its profile and neural bases in the nonfluent (nfvPPA) and logopenic (lvPPA) variants of primary progressive aphasia remain unclear. We aimed to characterize empathy at onset, examine its longitudinal course, and identify neural correlates in nfvPPA and lvPPA.

**METHODS:**

Thirty‐four nfvPPA, 38 lvPPA, and 45 control subjects completed neuropsychological and MRI assessment, with follow‐up up to 9 years. Empathy was measured using the Interpersonal Reactivity Index (Perspective Taking, Empathic Concern).

**RESULTS:**

At baseline, both PPA groups showed reduced cognitive empathy but preserved affective empathy. Lower empathy correlated with broader cognitive deficits in nfvPPA and predicted greater caregiver burden. Longitudinally, empathy remained stable in nfvPPA but declined in lvPPA. Brain–behavior analyses implicated temporo‐parietal and frontal‐insular regions, with progressive frontal atrophy predicting cognitive empathy decline in lvPPA.

**DISCUSSION:**

Findings demonstrate early empathy deficits in both variants, distinct trajectories, and syndrome‐specific neural substrates, with important clinical implications.

## Highlights

- Both primary progressive aphasia (PPA) variants show cognitive empathy deficits at baseline.
- Empathy is stable in nonfluent variant PPS (nfvPPA) but declines significantly in logopenic variant PPA (lvPPA) over time.
- Distinct brain networks support empathy in nfvPPA versus lvPPA.
- Progressive frontal atrophy predicts cognitive empathy decline in lvPPA.
- Empathy deficits predict greater caregiver burden.

## 1 BACKGROUND

Empathy is a multidimensional construct comprising affective empathy, the capacity to share and respond to another's emotional state, and cognitive empathy, the ability to understand another's perspective while preserving self–other boundaries.^1^ While social cognition has been increasingly recognized as a key diagnostic domain in major neurocognitive disorders such as Alzheimer's disease, frontotemporal dementia or Parkinson's disease,^2^, ^3^ it has been received less attention in rarer forms of dementias, including primary progressive aphasias.^4^, ^5^

Primary progressive aphasias (PPA) are progressive neurodegenerative disorders in which language is prominently affected at disease onset.^6^ This study focuses on the two non‐semantic variants: the nonfluent (nfvPPA) and logopenic (lvPPA) variants. The nfvPPA is characterized by agrammatism and effortful speech, with atrophy predominating in the left posterior fronto‐insular region and pathology usually consistent with tau‐positive frontotemporal lobar degeneration. The lvPPA presents with naming and repetition deficits with phonological errors. Atrophy mainly involves the left temporo‐parietal junction and is commonly associated with Alzheimer's disease.^6^

Although language impairment is the hallmark of PPA, increasing evidence points to concomitant non‐language deficits in memory, visuospatial abilities, attention, executive functions, and social cognition.^7^, ^8^, ^9^, ^10^ Within social cognition, studies have reported that affective empathy tends to be preserved in the early stages,^11^, ^12^, ^13^ but cognitive empathy may already be reduced in both nfvPPA and lvPPA.^12^, ^13^, ^14^ These empathy deficits affect caregiver well‐being^12^ and may be independent of language and cognitive impairments,^11^, ^12^ although further studies are needed to understand these complex relations. In addition, how empathy changes over time remains poorly understood.

Only one longitudinal study has investigated empathy in nfvPPA and lvPPA.^15^ Interestingly, it reported a decline in empathy over 1 year in both variants, more frequent in nfvPPA (60%) than lvPPA (33%). These findings suggest that the trajectory of empathy may aid differential diagnosis of these variants, which remain difficult.^16^ However, reliance on two questionnaire items in this study prevented distinction between cognitive and affective empathy.

Another unresolved issue concerns the neural substrates underlying empathy decline in PPA. Empathy relies on a distributed brain network, involving the medial prefrontal cortex, superior temporal sulcus, temporo‐parietal junction, and temporal poles for cognitive empathy, and the inferior frontal gyrus, inferior parietal lobe, anterior cingulate, and anterior insula for affective empathy.^17^ In PPA, only one study has investigated the neural substrates of empathy, showing negative correlations between cognitive empathy and cerebral metabolism in the parietal, insular, and frontal regions in lvPPA.^11^ The neural correlates of empathy in nfvPPA remain unknown, as do the relationships between longitudinal empathy decline and cortical thinning. Given that atrophy tends to extend posteriorly in nfvPPA and to anterior and contralateral regions in lvPPA,^10^, ^18^, ^19^ such progression is likely to impact both empathy components.

Taken together, these findings highlight important knowledge gaps regarding the nature (primary vs. secondary), the evolution, and the neural bases of empathy in PPA. This study therefore aimed to address these gaps by longitudinally assessing cognitive and affective empathy in nfvPPA and lvPPA. The first objective was to characterize empathy profiles at disease onset and their progression over time. We hypothesized that cognitive empathy would be impaired and affective empathy preserved in both variants at baseline, with steeper empathy decline in nfvPPA.^12^, ^15^ The second objective was to examine the relationships between empathy, language, cognition, and caregiver well‐being at baseline. We expected empathy deficits to be independent of language and cognitive impairments, and to predict caregiver burden.^11^, ^12^ The third objective was to identify the neural correlates of empathy at baseline and longitudinally. We hypothesized that cognitive empathy deficits would be associated with parietal, insular, and frontal changes in lvPPA, and anterior frontal and insular changes in nfvPPA, while affective empathy would involve frontal, parietal, anterior cingulate, and insular regions.^11^, ^17^

## 2 METHODS

### 2.1 Participants

A total of 115 individuals were included in the study (Table 1): 34 with nfvPPA, 38 with lvPPA, and 45 healthy controls (HCs). Participants’ records were extracted from the FRONTIER younger‐onset dementia research clinic database in Sydney, spanning assessments conducted between 2008 and 2024. Participants with PPA were followed annually, with most patients reassessed at least once, and a small subset followed beyond year 5. All available data were included up to the point of discontinuation.

**TABLE 1:** Demographic and clinical data for the participants with nfvPPA, lvPPA, and HCs at baseline assessment.

Parameter	nfvPPA	lvPPA	HC	F	*p*‐value	Post hoc tests
Sex (M/F)	19/15	19/19	22/23	0.416^b^	0.812	–
Age (years)	68 (10.7)	67.3 (7.0)	67.2 (6.6)	0.119	0.888	–
Education (years)	11.8 (2.7)	12.9 (3.2)	14.2 (2.6)	6.904	**0.001**	nfvPPA < HC
Disease duration (years)	3.78 (2.1)	3.97 (2.5)	–	644.5^c^	0.986	–
CDR‐FTLD SoB^a^	5.09 (3.8)	4.1 (2.9)	–	415.5^c^	0.362	–
No. of participants						
Baseline	34	38	45	–	–	–
Year 2	9	14	–	–	–	–
Year 3	4	9	–	–	–	–
Year 4	3	4	–	–	–	–
Year 5	2	2	–	–	–	–
Year 6‐9	1	2	–	–	–	–
Mean interval between assessment (months)	15.2 (5.9)	16.12 (12.3)		328.5^c^	0.978	–

*Note*: Bold font = significant results; Values are mean (standard deviation).

Abbreviations: CDR‐FTLD SoB, Clinical Dementia Rating Frontotemporal Lobar Degeneration Sum of Boxes scores; HC, healthy control; nfvPPA,  lvPPA, logopenic variant of primary progressive aphasia; nonfluent variant of primary progressive aphasia;

^a^ Missing data for two nfvPPA and eight lvPPA subjects.

^b^ Chi‐squared.

^c^ Mann–Whitney.

Diagnoses were established according to international criteria for PPA^6^ based on multidisciplinary consensus after clinical assessment, cognitive testing, structural MRI, and informant history. Diagnoses were confirmed at the latest available follow‐up. At baseline, the nfvPPA and lvPPA groups were matched for disease duration and functional disease severity, measured by the Clinical Dementia Rating Frontotemporal Lobar Degeneration Sum of Boxes score (CDR‐FTLD SoB)^20^ (Table 1).

**RESEARCH IN CONTEXT**

1. **Systematic review**: We reviewed literature on empathy in primary progressive aphasia (PPA) using PubMed and Google Scholar. While empathy deficits are recognized in major dementias, they remain understudied in PPA. Previous studies suggested preserved affective but impaired cognitive empathy in nonfluent (nfvPPA) and logopenic (lvPPA) variants at baseline, though longitudinal trajectories and neural substrates remained unclear.
2. **Interpretation**: Our longitudinal study shows both PPA variants have cognitive empathy loss at baseline, but trajectories diverge over time. Empathy remains stable in nfvPPA but declines in lvPPA. Brain–behavior analyses reveal distinct neural networks, left‐lateralized in nfvPPA versus bilateral in lvPPA, with progressive frontal atrophy driving decline in lvPPA.
3. **Future directions**: Empathy trajectory may aid differential diagnosis between variants. Future research should validate empathy assessment as a diagnostic tool, investigate links to functional outcomes, and examine associations with underlying pathology to clarify disease mechanisms.

HCs were recruited from the FRONTIER volunteer database and community sources. They showed no cognitive impairment (≥88/100 on the Addenbrooke's Cognitive Examination [ACE‐R or ACE‐III])^21^, ^22^, ^23^ and scored 0 on the Clinical Dementia Rating scale.^24^ They were age‐ and sex‐matched to the nfvPPA and lvPPA groups (*p* > 0.05) but had more years of education than the nfvPPA group (*p* = 0.001) (Table 1).

Exclusion criteria for all participants included neurological or psychiatric illness, substance abuse, or other neurodegenerative conditions. All participants (or their legal representative) provided informed consent. The study received ethics approval from the South Eastern Sydney Local Health District, the University of New South Wales, and the University of Sydney, and was conducted in accordance with the Declaration of Helsinki.

### 2.2 Materials

#### 2.2.1 Neuropsychological assessment

Participants completed a comprehensive neuropsychological battery covering multiple domains: global cognition (ACE‐R^22^ or ACE‐III,^21^), language (SYDBAT,^25^ naming, single‐word comprehension, semantic association, repetition), non‐verbal memory (3‐minute recall from the Rey Complex Figure [RCF]),^26^ attention and working memory (Digit Span forward and backward)^27^, and emotion recognition (Ekman 60 Faces Test – total score^28^ or FAST – total score,^29^, ^30^ with scores expressed as percentage accuracy for comparability).

#### 2.2.2 Empathy measure

Empathy was assessed using the Interpersonal Reactivity Index (IRI)^31^, a validated self‐ or informant‐report questionnaire composed of four subscales. In this study, only two subscales were used: Perspective Taking (PT), which assesses the cognitive ability to adopt another person's point of view (cognitive empathy), and Empathic Concern (EC), which reflects affective responses such as compassion and concern for others (affective empathy). These subscales are the most commonly used in studies of empathy and show strong psychometric properties.^14^, ^32^, ^33^ In contrast, the Fantasy (FS) and Personal Distress (PD) subscales have been associated with weaker construct validity and low relevance to empathy and social functioning, with PD also reflecting self‐oriented emotional reactivity rather than other‐oriented empathy.^32^

Each subscale includes seven items, rated on a 5‐point Likert scale (from 0 = does not describe at all to 4 = describes very well). Raw subscale scores (/28) were used for analysis, with higher scores indicating greater levels of empathy.

For participants with PPA, a close informant (e.g., spouse, adult child) completed a third‐person version of the IRI based on the participant's current behavior. This informant‐based approach accounts for reduced awareness commonly observed in neurodegenerative conditions^34^ and has been validated in prior studies of empathy in dementia.^32^, ^35^, ^36^ HCs completed the standard self‐report version, referring to the present moment.

#### 2.2.3 Carer burden and well‐being

Carer psychological well‐being and burden were assessed using two standardized questionnaires, completed by the primary informant of each participant with PPA. The Zarit Burden Interview (ZBI‐12 items;^37^) was used to evaluate perceived caregiving burden. Items are rated on a 5‐point Likert scale from 0 (never) to 4 (nearly always), with a maximum total score of 48. The total score reflects the overall impact of caregiving on emotional, physical, and social well‐being, with higher scores indicating greater burden.

The Depression Anxiety Stress Scales – 21 items (DASS‐21;^38^) were used to evaluate overall emotional distress. Items are rated on a 4‐point scale, covering symptoms of depression, anxiety, and stress. Total scores were multiplied by two to produce equivalent scores on the 42‐item version (maximum = 126), with higher scores indicating greater psychological distress.

### 2.3 MRI acquisition and pre‐processing

Whole brain structural magnetic resonance imaging (MRI) scans were acquired using a Philips Achieva or GE Discovery 3.0T scanner with a standard eight‐channel head coil. Two high‐resolution 3D T1‐weighted turbo field echo sequences were obtained with the following parameters: coronal orientation, matrix size 256 × 256, 200 slices, in‐plane resolution 1 mm^2^, slice thickness 1 mm, echo time/repetition time = 2.6/5.8 ms, and flip angle = 8°. Baseline MRI was available for 31 participants with nfvPPA, 36 with lvPPA, and 27 HCs. Longitudinal MRI follow‐up was conducted in 12 participants with nfvPPA and 13 with lvPPA, with annual MRI over 1 to 6 years (median = 2 scans). In total, 135 scans were included (50 nfvPPA, 58 lvPPA, 27 HCs). Scans were acquired within approximately 1 month of the behavioral assessment.

At each time point, the two T1‐weighted volumes were co‐registered and averaged to enhance signal‐to‐noise ratio and improve gray–white matter contrast. Cortical reconstruction and volumetric segmentation were performed using the recon‐all longitudinal pipeline in FreeSurfer (version 7.1.1), following established longitudinal processing pipelines^39^, ^40^, ^41^, ^42^, ^43^ and procedures.^44^ For each participant with multiple time points, an unbiased within‐subject template was first created using robust inverse consistent registration.^41^ Skull stripping, Talairach transformation, atlas registration, surface reconstruction, and parcellation were then initialized using information from the individual template to improve reliability and statistical sensitivity.^41^ Cortical thickness maps were smoothed using a 15 mm or 20 mm full‐width at half‐maximum Gaussian kernel to reduce inter‐subject variability and improve signal quality.^40^

### 2.4 Statistical analyses

#### 2.4.1 Behavioral analyses

All statistical analyses were performed using R (Version 2024.12.1 + 563;^45^). The significance level was set at *α *= 0.05 for all tests.

##### Baseline and longitudinal empathy analyses

Baseline empathy scores (IRI‐PT, IRI‐EC) were compared across the nfvPPA, lvPPA, and HC groups using analysis of covariance (ANCOVA) or ranked ANCOVA, depending on normality, with years of education as a covariate. Significant effects were followed by post‐hoc pairwise comparisons with Bonferroni correction.

Longitudinal changes in empathy (IRI‐PT and IRI‐EC) were examined in the nfvPPA and lvPPA groups using linear mixed‐effects (LME) models. Assumptions of linearity, normality, homoscedasticity, independence of residuals, and multicollinearity were assessed prior to modeling. Models included group (nfvPPA, lvPPA), time from baseline (in days), and their interaction as fixed effects, with participants as random effects. Significance of fixed effects and interactions was assessed using likelihood ratio tests. For significant interactions, post‐hoc comparisons of estimated marginal means (EMMs) were conducted using the *emmeans* package,^46^ with Bonferroni correction.

##### Associations between empathy, neuropsychological measures, and carer burden at baseline

Empathy and neuropsychological measures: Due to sample size constraints, simple linear regression models were used to examine the association between various neuropsychological measures (SYDBAT – naming, repetition, semantic fluency; ACE Total; RCF 3‐minute score; Digit Span forward and backward; Emotion recognition) and empathy scores (IRI‐PT and IRI‐EC). These analyses were performed separately for each PPA subgroup (nfvPPA and lvPPA).

Empathy and carer burden: Simple linear regression models were employed to assess the predictive relationship between IRI‐PT and IRI‐EC scores (as predictors) and total scores on the DASS and the ZBI (as dependent variables). These analyses were conducted on the combined patient group (nfvPPA and lvPPA), as caregiver impact was not expected to differ by diagnosis.

#### 2.4.2 Neuroimaging analyses

##### Baseline and longitudinal comparisons of cortical thickness

All imaging analyses were performed in FreeSurfer (v7.1.1). Statistical significance was set at *p* < 0.001 (uncorrected), with a conservative cluster‐extent threshold of *k* > 50 mm^2^, a commonly used approach in longitudinal PPA studies to minimize Type I error while maintaining sensitivity.^10^, ^18^, ^47^

Baseline comparisons of cortical thickness were conducted using whole‐brain vertex‐wise general linear models (GLM) with cortical thickness as the dependent variable and group (nfvPPA, lvPPA, HC) as the independent variable. Analyses included 31 individuals with nfvPPA, 36 with lvPPA, and 27 HCs (total = 94 scans).

Progressive cortical thinning was then examined using spatiotemporal LME models, implemented in Matlab with FreeSurfer tools,^48^ in participants with nfvPPA (12 participants, 31 scans) and lvPPA (13 participants, 34 scans). For each group, average trajectories of cortical thickness over time were inspected across the cortex. These showed approximately linear change, justifying the use of linear models. The spatiotemporal LME models included group (nfvPPA, lvPPA), time from baseline, and their interaction as fixed effects, and the intercept and time from baseline as random effects. The analyses tested whether cortical thickness changed significantly over time, and whether the rate of atrophy differed between diagnostic groups.

##### Associations between empathy and cortical thickness at baseline and longitudinally

At baseline, brain–behavior associations were assessed using whole‐brain vertex‐wise GLM to examine correlations between cortical thickness (dependent variable) and empathy scores (covariate) within each patient group (nfvPPA and lvPPA). Analyses focused only on empathy measures showing group differences at baseline (i.e., cognitive empathy, see the Results section). HCs were included in the models to increase variance and improve statistical power. Statistical maps were thresholded at *p* < 0.001 (uncorrected), with a cluster extent threshold of *k* > 50 mm^2^.

Longitudinal associations between cognitive empathy and cortical thickness were then examined in the lvPPA group (13 participants; 34 scans; see the Results section), as this was the only group showing longitudinal decline. Whole‐brain vertex‐wise GLM analyses were first conducted, but no significant clusters emerged, likely due to limited sample size. We therefore focused on eight a priori regions of interest (ROIs) to reduce the multiple‐comparisons burden. These were selected based on the empathy model proposed by Shamay‐Tsoory^17^ and on findings from Giacomucci et al.^11^, the only study to date investigating the neural bases of empathy in lvPPA. To further limit the number of ROIs, we retained only regions that showed associations with cognitive empathy at baseline. The final set of ROIs included: left ventromedial prefrontal cortex (mean of rostral anterior cingulate and medial orbitofrontal), left inferior parietal lobule (mean of inferior parietal and supramarginal; part of the temporo‐parietal junction), right temporal pole, left insula, bilateral middle frontal gyrus (mean of rostral and caudal middle frontal), and bilateral superior frontal gyrus. Mean cortical thickness values for each ROI were extracted in FreeSurfer using the Desikan–Killiany cortical atlas^49^ and exported to R (v2024.12.1). LME models were then applied to test whether baseline cortical thickness and its rate of change predicted empathy scores over time. Each model included ROI thickness, time from baseline, and their interaction as fixed effects, with a random intercept for participants. *p*‐Values were adjusted for multiple comparisons using false discovery rate (FDR) correction.

## 3 RESULTS

### 3.1 Baseline and longitudinal empathy analyses

At baseline, ANCOVA revealed a significant group effect on the IRI‐PT subscale (Table 2). Post‐hoc analyses showed lower scores in the nfvPPA group (*p* = 0.012) and the lvPPA group (*p* = 0.014) compared to HCs, with no difference between the two PPA groups (*p* > 0.05). No significant group differences were found for the IRI‐EC subscale (*p* > 0.05).

**TABLE 2:** IRI data of the participants with nfvPPA, lvPPA, and HCs at baseline assessment.

	nfvPPA	lvPPA	HC	F	*p*‐value	Post hoc tests
IRI – PT (/28)	13.8 (6.5)	14.0 (6.6)	17.6 (4.0)	5.837	**0.004**	nfvPPA = lvPPA < HC
IRI – EC (/28)	18.2 (7.6)	18.5 (6.4)	20.1 (4.6)	2.323^a^	0.103	–

*Note*. Bold font = significant results. Values are mean (standard deviation).

Abbreviations: ANCOVA, analysis of covariance; HC, healthy control; IRI‐EC, Interpersonal Reactivity Index – Empathic Concern (affective empathy); IRI‐PT, Interpersonal Reactivity Index – Perspective Taking (cognitive empathy); lvPPA, logopenic variant of primary progressive aphasia; nfvPPA, nonfluent variant of primary progressive aphasia.

^a^ Ranked ANCOVA.

**FIGURE 1:**
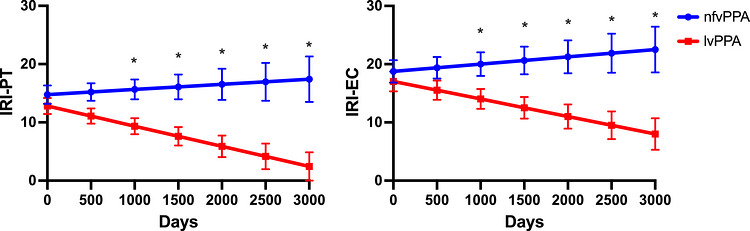
Predicted cognitive (IRI‐PT) and affective (IRI‐EC) empathy scores over time in the participants with nfvPPA and lvPPA. IRI‐EC, Interpersonal Reactivity Index – Empathic Concern; IRI‐PT, Interpersonal Reactivity Index – Perspective Taking; lvPPA, logopenic variant of primary progressive aphasia; nfvPPA, nonfluent variant of primary progressive aphasia.

For the longitudinal data, the linear mixed‐effects model for IRI‐PT revealed a significant main effect of time from baseline (*χ* ^2^(1) = 6.484, *p* < 0.02), indicating a decrease in IRI‐PT scores over time (*β* = ‐0.002, SE = 0.0008, *z* = ‐2.87, *p* < 0.01). A marginal main effect of Group was observed (*χ* ^2^(1) = 3.343, *p* = 0.068), with the lvPPA group showing significantly lower IRI‐PT scores compared to the nfvPPA group, independent of time from baseline (*β* = ‐4.158, SE = 1.94, *z* = ‐2.14, *p* < 0.05). Crucially, a significant Group × time from baseline interaction was present (*χ* ^2^(1) = 6.745, p < 0.01). This interaction indicated that the lvPPA group experienced a greater decline in IRI‐PT scores over time compared to the nfvPPA group. Predicted scores showed that the lvPPA group consistently had lower IRI‐PT scores than the nfvPPA group across all time points, and this disparity appeared to widen over the follow‐up period (Figure 1).

For the IRI‐EC subscale, the linear mixed‐effects model showed a significant main effect of time from baseline (*χ* ^2^(1) = 4.611, *p* < 0.04), demonstrating a decrease in IRI‐EC scores over time (*β* = −0.002, SE = 0.0008, *z* = ‐2.29, *p* < 0.03). The main effect of Group was not significant (*χ* ^2^(1) = 1.993, *p* = 0.158). However, a significant Group × time from baseline interaction was observed (*χ* ^2^(1) = 6.673, *p* < 0.01). This interaction indicated that the lvPPA group experienced a more pronounced decline in IRI‐EC scores over time compared to the nfvPPA group. Predicted scores revealed that the lvPPA group consistently exhibited lower IRI‐EC scores than the nfvPPA group across all time points, with the gap between groups widening over time (Figure 1).

### 3.2 Associations between empathy, neuropsychological measures, and carer burden at baseline

The full regression outputs (betas, standard errors, *t*‐values, *p*‐values, and *R* ^2^) are provided in the Supplementary Material (Tables S1–S3).

For the association between empathy and neuropsychological measures, results in the nfvPPA group showed that global cognition (ACE; *p *= 0.014, adjusted *R* ^2^ = 0.170), working memory (Digit Span backward; *p* = 0.023, adjusted *R* ^2^ = 0.157), and emotion recognition (*p* = 0.043, adjusted *R* ^2^ = 0.141) significantly predicted IRI‐PT scores. Working memory (Digit Span backward; *p* = 0.039, adjusted *R* ^2^ = 0.126) was the only significant predictor of IRI‐EC scores. In the lvPPA group, no cognitive variable significantly predicted IRI‐PT or IRI‐EC scores.

For the association between empathy and caregiver outcomes, lower IRI‐PT and IRI‐EC scores significantly predicted greater perceived burden (ZBI; *p* = 0.026 and *p* < 0.001, adjusted *R* ^2^ = 0.057 and 0.202, respectively). In addition, lower IRI‐EC scores predicted higher carer distress (DASS; *p* = 0.029, adjusted *R* ^2^ = 0.056).

### 3.3 Baseline and longitudinal comparisons of cortical thickness

Baseline analyses confirmed the expected atrophy patterns: nfvPPA showed left frontal thinning (inferior, middle, and superior frontal gyri, insula, precentral gyrus), while lvPPA showed predominant left temporoparietal involvement extending into temporal, parietal, and occipital cortices. Longitudinally, nfvPPA showed progressive frontal–parietal thinning, whereas lvPPA showed greater temporoparietal and insular decline. A detailed description of baseline and longitudinal atrophy patterns is provided in the Supplementary Material (Figure S1).

### 3.4 Associations between empathy and cortical thickness at baseline and over time

**FIGURE 2:**
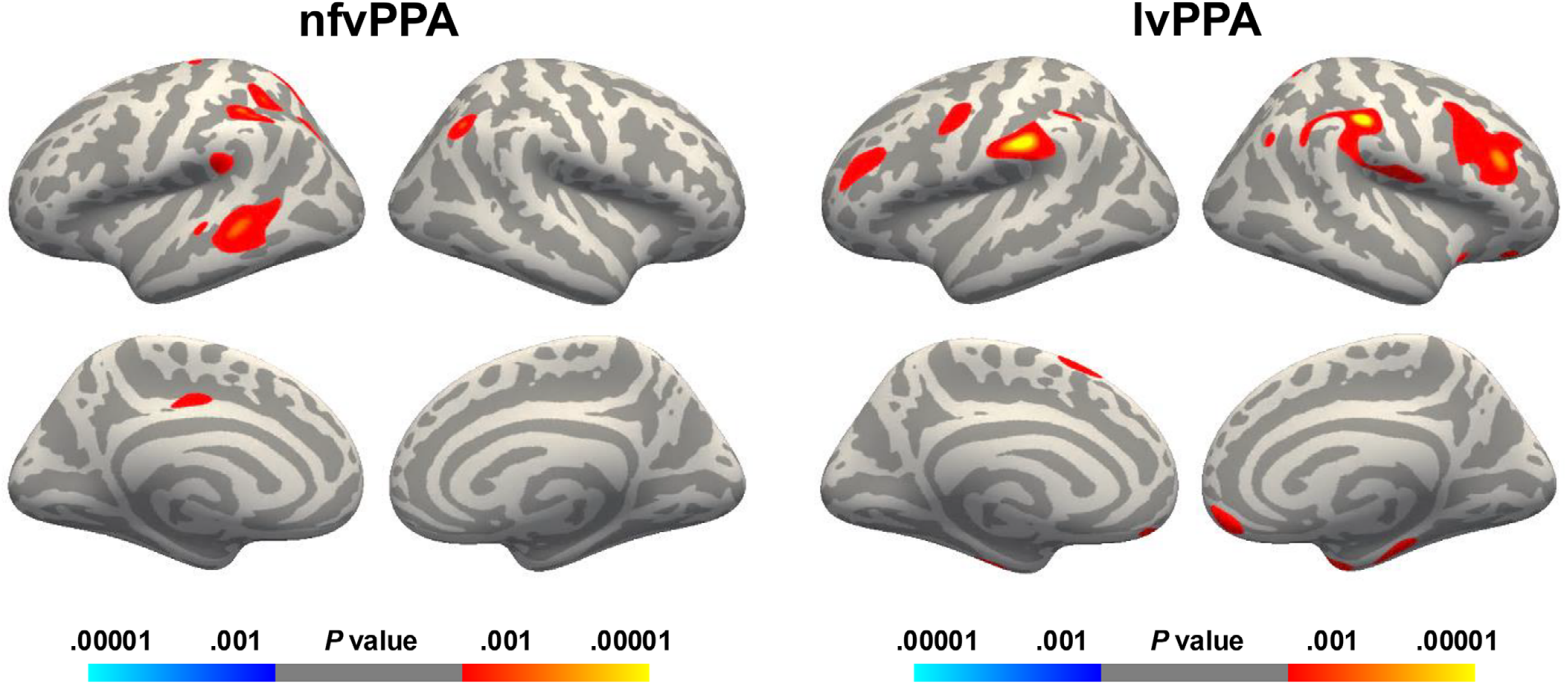
Associations between cognitive empathy (IRI‐PT) and cortical thickness at baseline. *Note*. Statistical maps smoothed at 20 mm, thresholded at p < 0.001 (uncorrected), with a conservative cluster‐extent threshold of k > 50 mm^2^. Red colors indicate positive correlations between cortical thickness and IRI‐PT scores. IRI‐PT, Interpersonal Reactivity Index – Perspective Taking; lvPPA, logopenic variant of primary progressive aphasia; nfvPPA, nonfluent variant of primary progressive aphasia.

Analyses focused on cognitive empathy (IRI‐PT), as this was the only measure to show group differences at baseline. Lower IRI‐PT scores in nfvPPA were linked to thinner cortical thickness in left‐lateralised regions, including frontal (precentral), parietal (supramarginal, superior and inferior parietal, posterior cingulate), and temporal cortices (middle and superior temporal gyri, banks of the superior temporal sulcus), with an additional cluster in the right inferior parietal cortex (Figure 2, left panel). In lvPPA, significant associations were more widespread and bilateral, involving frontal cortices (left precentral, superior frontal; right caudal middle frontal, pars opercularis, pars triangularis, lateral and medial orbitofrontal; bilateral rostral middle frontal), parietal regions (right inferior and superior parietal; bilateral postcentral and supramarginal), temporal regions (right fusiform, inferior temporal, temporal pole, entorhinal, parahippocampal), and the right insula (Figure 2, right panel).

**FIGURE 3:**
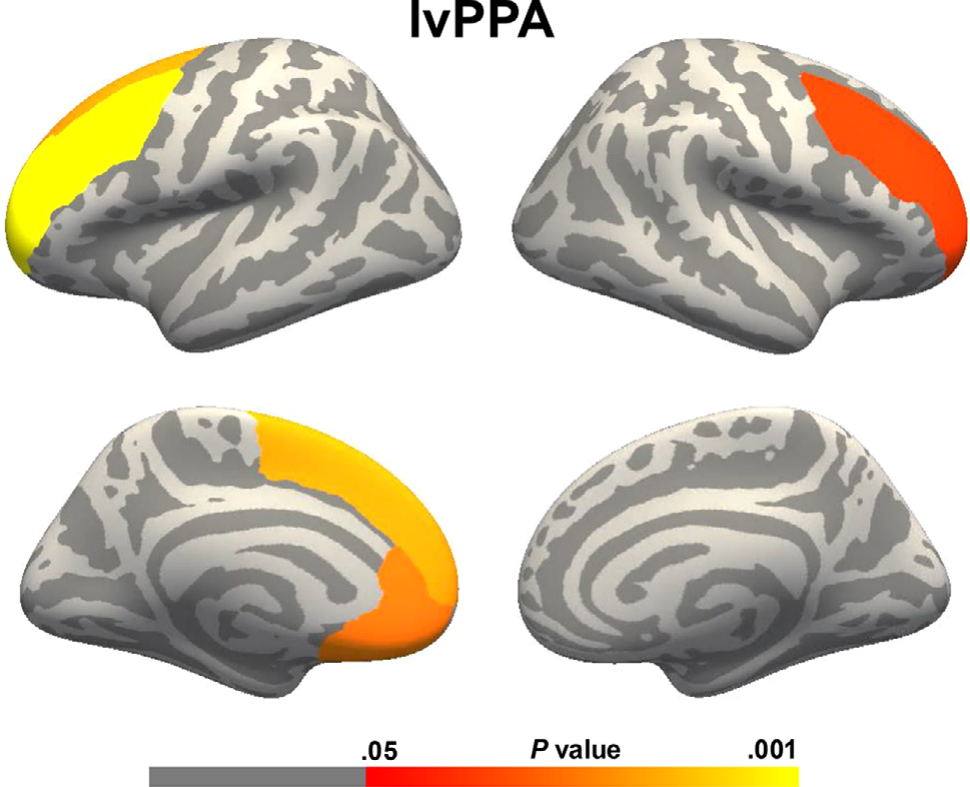
Associations between cognitive empathy (IRI‐PT) and cortical thickness over time in the participants with lvPPA. Cortical regions (ROIs) showing significant interaction with IRI‐PT in lvPPA, with thinning atrophy associated with lower cognitive empathy. Longitudinal results are thresholded at *p* = 0.05 corrected for multiple comparisons (FDR). FDR, false discovery rate; IRI‐PT, Interpersonal Reactivity Index – Perspective Taking; lvPPA, logopenic variant of primary progressive aphasia; ROI, region of interest.

Over time, only the lvPPA group showed a significant decline in empathy and was therefore included in longitudinal ROI analyses. Decline in IRI‐PT scores was associated with progressive cortical thinning in the bilateral middle frontal (left: *p* = 0.007; right: *p* = 0.031), the left superior frontal (*p* = 0.016) and the left ventromedial prefrontal cortices (*p* = 0.018) (Figure 3). A trend‐level association was also observed in the right superior frontal cortex (uncorrected *p* = 0.057; FDR‐corrected *p* = 0.091). No significant associations were found in the remaining ROIs.

Given that the lvPPA group also showed a significant decline in affective empathy, although without baseline deficits (see Results of empathy analyses), additional longitudinal ROI brain–behavior analyses were conducted using the same LME approach. ROIs were selected based on prior empathy literature,^17^, ^50^, ^51^ and included the left and right inferior frontal gyrus, anterior cingulate cortex, and insula, as well as the right inferior parietal lobule. These results are shown in the Supplementary Material (Table S4).

## 4 DISCUSSION

This study aimed to comprehensively evaluate cognitive and affective empathy at disease onset and over time in nfvPPA and lvPPA. Our longitudinal behavioral and neuroimaging analyses demonstrated distinct behavioral trajectories and divergent neural substrates of empathy in nfvPPA and lvPPA, providing new insights into empathy deficit mechanisms and progression in these syndromes.

### 4.1 Empathy at baseline: Similar profiles but different underlying mechanisms?

Both PPA groups showed impaired cognitive empathy but preserved affective empathy at baseline, aligning with previous studies^11^, ^12^, ^13^, ^14^. These findings confirm the presence of non‐language cognitive deficits from the early disease stages^8^ and reflect the fact that brain regions typically atrophied in PPA support both linguistic and non‐linguistic functions. Examination of the broad cognitive profile in these syndromes is therefore important, as it can facilitate early detection and refinement of the differential diagnosis across PPA variants.^52^

Although empathy deficits did not distinguish nfvPPA from lvPPA at baseline, our results suggest potentially different underlying mechanisms. In nfvPPA, both cognitive and affective empathy were associated with other cognitive deficits (global cognition, working memory, emotion recognition), suggesting that empathy difficulties may be related to broad cognitive impairments rather than reflecting a primary deficit in empathy. This pattern is consistent with prior work on emotion recognition showing that difficulties in nfvPPA often co‐occur with perceptual, attentional, or language‐related deficits.^53^, ^54^, ^55^, ^56^ However, the correlational nature of our findings precludes causal conclusions.

In contrast, the absence of associations between empathy and cognitive measures in our participants with lvPPA suggests a primary deficit of empathy in this variant. This finding converges with Giacomucci et al.^11^ and Hazelton et al.,^12^ who reported no significant correlations between empathy and standard neuropsychological tests assessing global cognition, language, memory, attention, executive functions, and emotion recognition in this variant.

Finally, our results demonstrated that empathy deficits negatively affect caregiver well‐being, echoing previous findings in PPA and other dementias.^12^, ^57^, ^58^, ^59^ Empathy enables relationship reciprocity within shared emotional environments.^60^ Social–cognitive changes are perceived early by spouses of PPA individuals, sometimes before language difficulties^61^ and, therefore, may affect caregiver well‐being at an early stage, with clinical implications (see below).

### 4.2 Empathy over time: When differences appear

Although empathy deficits did not differ between individuals with nfvPPA and lvPPA at baseline, their trajectories appeared to diverge over time. Empathy remained relatively stable in nfvPPA, whereas individuals with lvPPA showed a greater decline in both cognitive and affective empathy. These findings contrast with van Langenhove et al.,^15^ who reported higher empathy loss in nfvPPA than in lvPPA. Methodological differences likely account for this discrepancy. Specifically, van Langenhove and colleagues^15^ used only two items from a global behavioral questionnaire and focused on the proportions of individuals showing change over a 1‐year period. In our study, predicted scores suggest the empathy gap may widen over time. Therefore, the pronounced empathy decline in lvPPA may not have been captured by their less sensitive measures and shorter timeframe.

This greater decline in lvPPA is consistent with findings from other cognitive domains, where this variant has been shown to deteriorate more rapidly than nfvPPA.^8^ These observations, however, should be interpreted with caution given the limited longitudinal sample. Further studies are needed to clarify empathy trajectory, as this feature could contribute to the differential diagnosis of the two variants.

### 4.3 Neural substrates of empathy

Baseline brain–behavior analyses confirmed cognitive empathy depends on distributed neural networks, partly shared yet distinct across the two PPA variants. In both PPA groups, IRI‐PT scores were associated with the inferior parietal lobule and temporo‐parietal junction, two key hubs of cognitive empathy,^62^ previously linked to IRI‐PT in lvPPA.^11^ The involvement of the temporo‐parietal junction, along with the superior temporal sulcus in nfvPPA and the prefrontal cortex and temporal pole in lvPPA, further highlights the role of theory of mind in cognitive empathy, especially perspective taking.^17^

In contrast to the left‐lateralized pattern in nfvPPA, lvPPA showed more bi‐hemispheric associations. Prior studies have emphasized a stronger contribution of the right hemisphere to cognitive empathy in individuals with mild cognitive impairment,^63^ Alzheimer's disease,^11^, ^64^ and other neurodegenerative diseases,^32^ suggesting a link with more severe empathy deficits.^65^ The involvement of both the left‐ and right‐hemisphere regions in lvPPA likely reflects the language‐based nature of the syndrome as well as potentially more severe empathy deficits, as indicated by the longitudinal decline observed only in this variant.

In lvPPA, brain regions associated with cognitive empathy were also widespread, involving the right insula and several frontal regions. The insula, central to empathy,^62^, ^66^ contributes through interoceptive processing^67^, ^68^ and salience network functions,^69^ integrating sensory, emotional, and bodily signals to coordinate brain dynamics. Its link with a cognitive empathy measure in our study suggests closer coupling of empathy components in lvPPA, perhaps reflecting disease spread or compensatory reliance on embodied emotion.

The dorsolateral, orbitofrontal, and ventromedial prefrontal cortices bilaterally, as well as the right inferior frontal gyrus, were also strongly associated with IRI‐PT in lvPPA. This central role of the frontal lobe in cognitive empathy aligns with previous findings in healthy individuals^51^, ^70^ and individuals with various neurodegenerative diseases.^32^, ^64^, ^71^, ^72^ The dorsolateral prefrontal cortex appears particularly involved in higher‐level forms of empathy, such as perspective taking.^62^ The ventromedial prefrontal cortex has also been identified as a key hub of the cognitive empathy network,^17^ notably contributing to self‐other distinction, a core dimension in the conceptualization of empathy.^1^ Finally, these frontal regions, especially the inferior frontal gyrus, are strongly engaged in the mirror neuron system, which supports the understanding of others’ intentions by mirroring their actions and emotional states.^73^

Interestingly, progressive cortical thinning in these frontal regions, especially in the bilateral middle frontal, left superior frontal, and left ventromedial prefrontal cortices, was significantly associated with a decline in cognitive empathy over time in lvPPA. These findings add a longitudinal dimension to our baseline results, suggesting that the functional integrity of these frontal structures play an important role in maintaining cognitive empathy. One possible interpretation is that frontal regions are increasingly recruited to support empathy as posterior regions degenerate, consistent with the widespread bilateral network atrophy already present at baseline in our participants with lvPPA. As cortical thinning in these frontal areas is already associated with reduced empathy, such compensatory recruitment may be only partial or ultimately insufficient. With the progression of atrophy into these frontal hubs, the loss of their supportive role may contribute to the worsening of empathy in this patient group.

### 4.4 Clinical implications

Our findings have important clinical implications. The observation of empathy loss at baseline reinforces the importance of considering the full cognitive profiles, and not only language functions, when diagnosing PPA.^74^ This broader perspective may contribute to earlier diagnosis, which remains a challenge,^75^ and improve the accuracy of differential diagnosis. Distinguishing nfvPPA from lvPPA can be difficult due to overlapping language deficits^76^, ^77^; in this context, empathy assessment may prove valuable for longer‐term differential diagnosis, particularly as the language and cognitive profiles become more mixed and severe.

Accounting for the full symptom profile also has therapeutic relevance. Given the impact of empathy loss on caregiver well‐being, already evident in early disease stages, raising awareness and providing education about socio‐cognitive changes is crucial. Early psychoeducation can reduce caregiver burden, which is critical in dementia care pathways.

### 4.5 Limitations

Our study has limitations. Although our baseline sample was relatively large for this population, longitudinal data (particularly beyond 3 years) were more limited, reducing statistical power. As such, longitudinal findings need to be interpreted with caution and considered preliminary. While the observed increase in the gap between nfvPPA and lvPPA over time is of interest, it will require replication in larger longitudinal cohorts before firm conclusions can be drawn.

Sample size also constrained our longitudinal brain–behavior analyses. Limited statistical power required ROIs selection rather than whole‐brain analyses. Although our ROI choice was supported by literature, this approach may have masked additional brain regions involved in empathy change in PPA.

Another limitation is the inclusion of only two PPA variants (nfvPPA and lvPPA). While this choice limits the generalizability of our findings across the full PPA spectrum, it was made deliberately. In contrast to svPPA which has been extensively studied in relation to socio‐emotional impairments,^4^ nfvPPA and lvPPA remain comparatively underexplored. Focusing on these variants was therefore intended to provide additional insights into empathy changes and their potential clinical relevance, particularly given the challenges in distinguishing between them.^5^, ^16^

## 5 CONCLUSIONS

This study is the first to investigate the trajectory, underlying mechanisms, and neural substrates of empathy in nfvPPA and lvPPA. We confirmed early cognitive empathy deficits in both variants, which appear secondary to other cognitive impairments in nfvPPA but primary in lvPPA. A decline in both cognitive and affective empathy seems to characterize disease progression in lvPPA. We also highlighted the involvement of a temporoparietal network in cognitive empathy, with a stronger left‐lateralization in nfvPPA and a broader extension into frontal regions in lvPPA. Frontal atrophy, in particular, appears central to the decline of cognitive empathy in lvPPA. These findings fill critical gaps in understanding empathy in these rarer forms of dementia and have important implications for diagnosis and therapy.

## CONFLICT OF INTEREST STATEMENT

The authors report there are no competing interests to declare. Author disclosures are available in the supporting information.

## CONSENT STATEMENT

All human subjects provided informed consent.

## Supporting information




**Supporting Information**: alz71519‐sup‐0001‐SuppMat.docx

**Supporting Information**: alz71519‐sup‐0002‐SuppMat.pdf
