# Supplementary material for: Empathy in primary progressive aphasia: Neural signatures and longitudinal trajectories
Source: Alzheimers Dement. 2026 May 25;22(5):e71519. doi: 10.1002/alz.71519 (PMC13240075; doi:10.1002/alz.71519)
Supplement: Supplementary file 1 — Supporting Information: alz71519‐sup‐0001‐SuppMat.docx [file ALZ-22-e71519-s001.docx]

**Supplementary material**

***Associations between empathy, neuropsychological measure and carer burden at baseline***

**Table S1.** Coefficients from the simple linear regressions predicting cognitive empathy (IRI-PT) in the nfvPPA and lvPPA participants

| **Predictors** | **Group** | **B** | **SE** | **t** | **p-value** | **R2** | **Adj_R2** |
| --- | --- | --- | --- | --- | --- | --- | --- |
| SYDBAT – naming | nfvPPA | 0.048 | 0.193 | 0.247 | .807 | 0.002 | -0.035 |
|  | lvPPA | -0.239 | 0.171 | -1.397 | .172 | 0.056 | 0.027 |
| SYDBAT – Repetition | nfvPPA | -0.034 | 0.128 | -0.263 | .794 | 0.003 | -0.036 |
|  | lvPPA | 0.026 | 0.226 | 0.114 | .910 | 0.000 | -0.030 |
| SYDBAT – Semantic | nfvPPA | 0.462 | 0.367 | 1.261 | .218 | 0.052 | 0.019 |
|  | lvPPA | 0.276 | 0.314 | 0.879 | .386 | 0.024 | -0.007 |
| ACE Total | nfvPPA | 0.174 | 0.066 | 2.634 | **.014** | 0.199 | 0.170 |
|  | lvPPA | -0.042 | 0.082 | -0.517 | .609 | 0.008 | -0.022 |
| RCF | nfvPPA | 0.387 | 0.223 | 1.738 | .095 | 0.108 | 0.072 |
|  | lvPPA | 0.130 | 0.156 | 0.834 | .411 | 0.023 | -0.010 |
| Digit Span forward | nfvPPA | 0.352 | 1.090 | 0.323 | .749 | 0.004 | -0.036 |
|  | lvPPA | -0.566 | 0.801 | -0.706 | .485 | 0.015 | -0.015 |
| Digit Span backward | nfvPPA | 2.877 | 1.192 | 2.414 | **.023** | 0.189 | 0.157 |
|  | lvPPA | 1.274 | 1.032 | 1.234 | .226 | 0.045 | 0.016 |
| Emotion recognition | nfvPPA | 0.162 | 0.075 | 2.149 | **.043** | 0.180 | 0.141 |
|  | lvPPA | 0.000 | 0.077 | -0.002 | .998 | 0.000 | -0.033 |

*Note.* Bold font = significant results

**Table S2.** Coefficients from the simple linear regressions predicting affective empathy (IRI-EC) in the nfvPPA and lvPPA participants

| **Predictors** | **Group** | **B** | **Beta** | **t** | **p-value** | **R2** | **Adj_R2** |
| --- | --- | --- | --- | --- | --- | --- | --- |
| SYDBAT – naming | nfvPPA | -0.045 | 0.203 | -0.223 | .825 | 0.002 | -0.035 |
|  | lvPPA | 0.011 | 0.161 | 0.065 | .948 | 0.000 | -0.030 |
| SYDBAT – Repetition | nfvPPA | 0.175 | 0.129 | 1.359 | .186 | 0.066 | 0.030 |
|  | lvPPA | 0.159 | 0.205 | 0.780 | .441 | 0.018 | -0.012 |
| SYDBAT – Semantic | nfvPPA | 0.413 | 0.411 | 1.004 | .324 | 0.034 | 0.000 |
|  | lvPPA | -0.247 | 0.287 | -0.861 | .396 | 0.023 | -0.008 |
| ACE Total | nfvPPA | 0.131 | 0.082 | 1.601 | .121 | 0.084 | 0.051 |
|  | lvPPA | -0.022 | 0.075 | -0.300 | .766 | 0.003 | -0.028 |
| RCF | nfvPPA | 0.305 | 0.283 | 1.078 | .291 | 0.044 | 0.006 |
|  | lvPPA | -0.013 | 0.143 | -0.089 | .930 | 0.000 | -0.033 |
| Digit Span forward | nfvPPA | 0.651 | 1.195 | 0.544 | .591 | 0.012 | -0.028 |
|  | lvPPA | 0.083 | 0.731 | 0.113 | .911 | 0.000 | -0.031 |
| Digit Span backward | nfvPPA | 2.908 | 1.336 | 2.177 | **.039** | 0.159 | 0.126 |
|  | lvPPA | 0.143 | 0.957 | 0.149 | .882 | 0.001 | -0.031 |
| Emotion recognition | nfvPPA | 0.100 | 0.088 | 1.128 | .272 | 0.057 | 0.012 |
|  | lvPPA | 0.002 | 0.076 | 0.022 | .983 | 0.000 | -0.033 |

*Note.* Bold font = significant results

**Table S3.** Coefficients from the simple linear regressions predicting carer burden (ZBI, DASS) in the nfvPPA and lvPPA participants

| **Predictors** | **B** | **Beta** | **t** | **p-value** | **R2** | **Adj_R2** |
| --- | --- | --- | --- | --- | --- | --- |
| ***DASS*** | | | | | | |
| IRI-PT | -0.232 | 0.304 | -0.762 | .449 | 0.009 | -0.006 |
| IRI-EC | -0.646 | 0.289 | -2.237 | **.029** | 0.070 | 0.056 |
| ***ZBI*** | | | | | | |
| IRI-PT | -0.336 | 0.148 | -2.268 | **.026** | 0.070 | 0.057 |
| IRI-EC | -0.556 | 0.129 | -4.302 | **< .001** | 0.214 | 0.202 |

*Note.* Bold font = significant results

***Associations between cognitive and affective empathy and cortical thickness over time in the participants with lvPPA***

**Table S4.** Full model outputs for the ROIs of the cognitive (IR-PT) and affective (IRI-EC) empathy in the lvPPA participants

| **ROI** | **β** | **SE** | **df** | **t value** | **p-value** | **p-value FDR** |
| --- | --- | --- | --- | --- | --- | --- |
| ***Cognitive empathy*** |  |  |  |  |  |  |
| LH middle frontal | -7.072 | 1.874 | 24.494 | -3.773 | **<.001** | **.007** |
| LH superior frontal | -6.260 | 2.000 | 28.381 | -3.130 | **.004** | **.016** |
| LH vmPFC | -5.978 | 2.016 | 23.095 | -2.966 | **.007** | **.018** |
| RH middle frontal | -7.642 | 2.938 | 23.729 | -2.601 | **.016** | **.031** |
| RH superior frontal | -6.750 | 3.376 | 24.245 | -1.999 | *.057* | .091 |
| RH temporal pole | -1.455 | 0.949 | 26.752 | -1.533 | .137 | .157 |
| LH insula | -2.319 | 1.503 | 22.155 | -1.543 | .137 | .157 |
| LH IPL | -6.750 | 3.376 | 24.245 | -1.999 | .456 | .456 |
| ***Affective empathy*** |  |  |  |  |  |  |
| RH insula | 4.015 | 3.169 | 21.474 | 1.267 | .219 | .774 |
| LH insula | 1.077 | 1.816 | 21.399 | 0.593 | .559 | .774 |
| RH inferior frontal | 1.613 | 3.498 | 22.627 | 0.461 | .649 | .774 |
| LH inferior frontal | 0.9114 | 1.9842 | 22.5908 | 0.459 | .650 | .774 |
| LH anterior cingulate | -2.759 | 6.195 | 23.726 | -0.445 | .660 | .774 |
| RH inferior parietal | -1.019 | 3.089 | 23.596 | -0.330 | .744 | .774 |
| RH anterior cingulate | -0.870 | 2.995 | 22.662 | -0.291 | .774 | .774 |

*Note.* Bold font = significant results; italic font = marginally significant results; IPL = inferior parietal lobule; vmPFC = ventromedial prefrontal cortex

***Baseline and longitudinal comparisons of cortical thickness***

**Figure S1.** Baseline and longitudinal patterns of cortical thinning in nfvPPA and lvPPA

*Note.* The left panels display baseline cortical thinning for each PPA group relative to controls (top and middle) and between-group contrasts (bottom). The right panels show longitudinal changes within groups (top and middle) and direct comparisons across groups (bottom). Statistical maps were smoothed with a 15 mm Gaussian kernel for baseline analyses and 20 mm for longitudinal analyses, and thresholded at p < .001, with a cluster extent threshold of k > 50 mm² for all tests.

At baseline, the nfvPPA group showed predominantly left frontal atrophy, affecting the inferior frontal gyrus, orbitofrontal cortex, superior and middle frontal gyri, precentral gyrus, and insula, with additional involvement of the left supramarginal gyrus (Figure S1, left panel). Milder but corresponding thinning was also observed in the right frontal lobe. In contrast, the lvPPA group showed a widespread, left-lateralized pattern centred on the temporoparietal junction, extending through the superior, middle, and inferior temporal gyri, fusiform, parahippocampal, inferior and superior parietal lobes, precuneus, posterior cingulate, and lateral occipital cortex. Frontal and insular involvement was also present, with less extensive but bilateral thinning in homologous right hemisphere regions. Direct comparisons confirmed that lvPPA showed significantly greater atrophy than nfvPPA in posterior temporoparietal and occipital cortices, with no regions where nfvPPA showed greater thinning than lvPPA.

Longitudinal changes within and between syndromes are shown in Figure S1 (right panel). In nfvPPA, cortical thinning progressed within regions already affected at baseline, including the left inferior frontal gyrus, middle and superior frontal cortices, insula, precentral and postcentral gyri, and anterior cingulate. Atrophy also extended posteriorly into the left superior parietal, precuneus, and posterior cingulate, with widespread mirroring in the right hemisphere involving frontal, temporal, parietal, and occipital cortices. In lvPPA, progressive thinning was most pronounced in the left temporoparietal cortex, encompassing superior, middle, and inferior temporal regions, inferior and superior parietal cortices, posterior cingulate, precuneus, and lingual gyrus, with additional decline in left frontal and insular regions. Smaller clusters of atrophy were also observed in homologous right temporal, parietal, and frontal regions. Direct group comparisons revealed that nfvPPA showed greater rates of thinning in left superior frontal regions, whereas lvPPA showed greater decline in temporo-parietal and insular cortices.
